# Dendritic Cell Regulation of Graft-Vs.-Host Disease: Immunostimulation and Tolerance

**DOI:** 10.3389/fimmu.2019.00093

**Published:** 2019-02-01

**Authors:** Hongshuang Yu, Yuanyuan Tian, Ying Wang, Shin Mineishi, Yi Zhang

**Affiliations:** ^1^Fels Institute for Cancer Research and Molecular Biology, Temple University, Philadelphia, PA, United States; ^2^Department of Medicine, Pennsylvania State University, Hershey, PA, United States; ^3^Department of Microbiology & Immunology, Temple University, Philadelphia, PA, United States

**Keywords:** graft-vs.-host, disease, dendritic cells, transcription factors, alloreactive T cells, immunostimulation, immune tolerance

## Abstract

Graft-vs.-host disease (GVHD) remains a significant cause of morbidity and mortality after allogeneic hematopoietic stem cell transplantation (allo-HSCT). Significant progresses have been made in defining the dichotomous role of dendritic cells (DCs) in the development of GVHD. Host-derived DCs are important to elicit allogeneic T cell responses, whereas certain donor-types of DCs derived from newly engrafted hematopoietic stem/progenitor cells (HSPCs) can amply this graft-vs.-host reaction. In contrast, some DCs also play non-redundant roles in mediating immune tolerance. They induce apoptotic deletion of host-reactive donor T cells while promoting expansion and function of regulatory T cells (Treg). Unfortunately, this tolerogenic effect of DCs is impaired during GVHD. Severe GVHD in patients subject to allo-HSCT is associated with significantly decreased number of circulating peripheral blood DCs during engraftment. Existing studies reveal that GVHD causes delayed reconstitution of donor DCs from engrafted HSPCs, impairs the antigen presentation function of newly generated DCs and reduces the capacity of DCs to regulate Treg. The present review will discuss the importance of DCs in alloimmunity and the mechanism underlying DC reconstitution after allo-HSCT.

## Introduction

Allogenic hematopoietic stem cell transplantation (allo-HSCT) is a potentially curative therapy for many hematological malignancies, such as leukemia, lymphoma, and multiple myeloma (1, 2). This beneficial effect is largely derived from infused donor immune cells that can eliminate malignant cells, a process known as graft-vs.-leukemia (GVL) response (3–5). However, the success of the procedure is limited by the life-threatening complication graft-vs.-host disease (GVHD), in which the gastrointestinal (GI) tract, skin and liver are preferentially damaged (2, 6–9).

GVHD is mediated by infused donor T cells that recognize and react to histocompatibility differences between the host and donor (9–12). Host-derived antigen-presenting cells (APCs) can directly present antigens to prime allogenic donor T cells, whereas donor-derived APCs can present host antigens to donor T cells via indirect antigen presentation (10, 12, 13). Initial studies demonstrate that host APCs are critical for donor CD8^+^ T cell-mediated GVHD. Subsequent studies indicate that either host or donor APCs are sufficient to induce CD4^+^ T cell-dependent GVHD (9–12, 14–17). Importantly, unlike T cell responses to pathogens in which hematopoietic APCs prime T cells, alloreactive T cell responses in the setting of allo-HSCT may be primed by both hematopoietic and non-hematopoietic APCs (9–12, 14–17).

DCs are the most potent professional APCs known to elicit primary T cell responses (18–20). Based on their surface phenotype, anatomical location and function, DCs at the steady state are broadly categorized into conventional DCs (cDCs) and plasmacytoid DCs (pDCs). Under inflammatory condition, both DC subsets undergo profound changes in their phenotype and functionality (21–25). For example, in response to inflammatory stimuli, DCs may be primed selectively to produce special types of cytokines (e.g., IL-12, IL-23) and Notch ligands (e.g., Delta-like 1 (DLL1) and DLL4). These DC-derived molecules are important to instruct antigen-activated T cells to differentiate into distinct lineages of effector T cells, such as T helper (TH)1, TH17 cells, and cytotoxic T cells (CTLs) (26–33).

Over the past two decades, both clinical and preclinical studies have demonstrated dichotomous roles of DCs in GVHD (9, 34, 35). While some DCs induce alloreactive T cell responses mediating host tissue injury, other DC subsets induce donor T cell tolerance against the host tissue. In this review, we will discuss these effects of DC-mediated immunogenicity and tolerogenicity during GVHD.

## DC Induction of GVHD

DCs are specialized APCs that play non-redundant roles in regulating both immunity and tolerance (9, 18, 36–44). In the setting of allo-HSCT, host-derived DCs are important for donor T cell-mediated GVHD in the liver, colon and skin (9–12, 16, 17, 35, 45). *De novo* generated donor APCs, including DCs, are also required to induce maximal GVHD through a complex mechanism (9–11, 35).

### Host DCs and Initiation of Alloreactive T Cell Responses

Shlomchik and colleagues demonstrate, for the first time, that host hematopoietic APCs are critical for induction of the disease, and donor APCs can mediate maximal GVHD (10, 12). Subsequent studies reveal that host DCs, which are activated during preparative conditioning for allo-HSCT, present host antigens to prime donor CD4^+^ and CD8^+^ T cells and promote their proliferation and differentiation into alloreactive effector cells (17, 46). Add-back of WT host-type cDCs or pDCs causes severe GVHD in mice lacking MHC class-I or MHC class-II, respectively (47), further strengthening the importance of host DCs in mediating GVHD (Table 1). However, these studies do not explain whether host DCs contribute to GVHD when all the other types of host APCs, including B cells, macrophages and non-hematopoietic APCs, are intact. For example, host B cells produced high levels of IL-10 to modulate alloreactive T cell responses *in vivo* (57), Recipient macrophages, which resist the conditioning regimen, persisted in patients for several weeks following allo-HSCT and limited the severity of GVHD (58). In contrast, non-hematopoietic APCs activated by irradiation induce potent allo-specific responses in peripheral tissues(14, 59).

**Table 1:** Effect of different DC subsets in GVHD.

**DC subset**	**Effect on GVHD**	**Origin**	**Method**	**GVHD model**	**Outcome**	**Mechanism**	**Authors/References**
cDCs or pDCs	Induction	Host	Add-back of cDCs or pDCs	Balb/c → B6 AKR → C3H	GVHD↑	Prime allo-T cell response	Koyama et al. (47)
LCs	Induction	Host	Depletion of LCs	B6 → Balb/c	skin GVHD↓	Increase donor T cell infiltrating in the skin	Merad et al. (40)
LCs	No effect	Host	Depletion of LCs	B6 → Balb/c C3H → B6	GVHD maintained	-	Li et al. (48)
cDCs	Induction	Donor	Depletion of CD11c^+^ cDCs	B6 → Balb/c C3H → B6 B6 → B6D2F1	GVHD↓	Inhibit donor T cell proliferation	Markey et al. (49)
CD103^+^ CD11b^−^DCs	Induction	Donor	Depletion of CD103^+^CD11b^−^ cDCs	B6 → Balb/c	GVHD↑	Induce expansion and differentiation of donor T cells within the mLNs	Koyama et al. (13)
CD8α^+^ DCs	Tolerance	Host	Depletion of CD8^+^ cDCs	Balb/c → B6	GVHD↑	Reduce numbers of Tregs and TGF- β levels	Weber et al. (50)
CD8α^+^ DCs	Tolerance	Host	Pre-treatment of the recipient with Flt3L	B6 → B6D2F1	GVHD↓	Suppress donor T cell responses to host antigens	Teshima et al. (41)
CD8α^+^ DCs	Tolerance	Host	Pre-treatment of recipients with Flt3L	C3H → B6 B6 → B6D2F1	GVHD↓	Functionally delete of the alloreactive T-cell	Markey et al. (51)
CD8α^+^ cDCs	No effect	Host	Depletion of CD8^+^ cDCs	C3H → B6	GVHD maintained	-	Toubai et al. (52)
CCR9^+^ pDCs	Tolerance	Host	Transfer of CCR9^+^ pDCs	Balb/c → B6	GVHD↓	Promote Treg expansion and function Suppress antigen-specific T responses	Hadeiba et al. (53)
SAHA treated moDCs	Tolerance	Host	Transfer of moDC treated with SAHA	Balb/c → B6	GVHD↓	Promote Treg expansion and function	Reddy et al. (54)
pre-pDCs	Tolerance	Donor	Depletion of pre-pDCs from BM grafts	B6 → Balb/c B6 → B6D2F1	GVHD↑	Inhibit T cell proliferation in a contact-dependent fashion	Banovic et al. (55)
pre-pDCs	Tolerance	Donor	Transfer of BM pre-pDCs	B6 → B10	GVHD↓	Increase Tregs and decrease alloreactive effector T cells	Lu et al. (56)

The role of host DCs in the development of GVHD in the presence of functional macrophages and non-hematopoietic APCs has been studied by several groups. Merad et al. examined the role of host Langerhans cells (LCs), a distinct subset of DCs located in the skin (19), in cutaneous GVHD (40). Administration of donor T cells to bone marrow (BM)-chimeric mice with persistent host LCs, but not to mice whose LCs had been replaced, resulted in marked skin GVHD (40), suggesting that host LCs are important for mediating the disease in the skin. Intriguingly, other studies show that LCs were dispensable for the induction of skin GVHD (48). In one of those studies, donor T cells and BM cells were transferred into lethally irradiated transgenic recipient mice in which epidermal LCs expressed the Diphtheria toxin A (DTA) under the control of the human Langerin locus (48). Deficiency of LCs did not affect the development of either CD8^+^ T cell- or CD4^+^ T cell-mediated GVHD (48). How to reconcile these observations remains controversial.

### Donor DCs Amplify GVH Reaction by Cross-Presenting Host-Type Antigen

In the setting of allo-HSCT, *de novo* generated donor APCs are also found to be important for GVHD (9–11, 35). Studies by Markey et al. suggested that donor cDCs isolated from the spleen were the most effective population in presenting alloantigens and stimulating naïve donor T cell responses early after allo-HSCT (49). Intriguingly, upon exposure to GVH inflammation, donor CD103^+^CD11b^−^ cDCs, which are independent of the transcription factor IRF4 for their development (60, 61), captured alloantigen in the colon and migrated into the mesenteric lymph node to amplify alloreactive T cell responses (13). This suggests that tissue resident DCs may play important roles in regulating GVH reactions, which is supported by our early studies. We found that selective depletion of both host- and donor-type APCs, including DCs, in visceral organs led to significantly reduced GVHD in the liver but not in the skin (11). These observations suggest that donor DCs possess great capacity to orchestrate the alloreactive T cell response both in the lymphoid organ and non-lymphoid tissues, eliciting different types of GVHD.

### DC-Derived IL-12 and Notch Ligands Shape Alloreactive T Cell Responses

**Figure 1:**
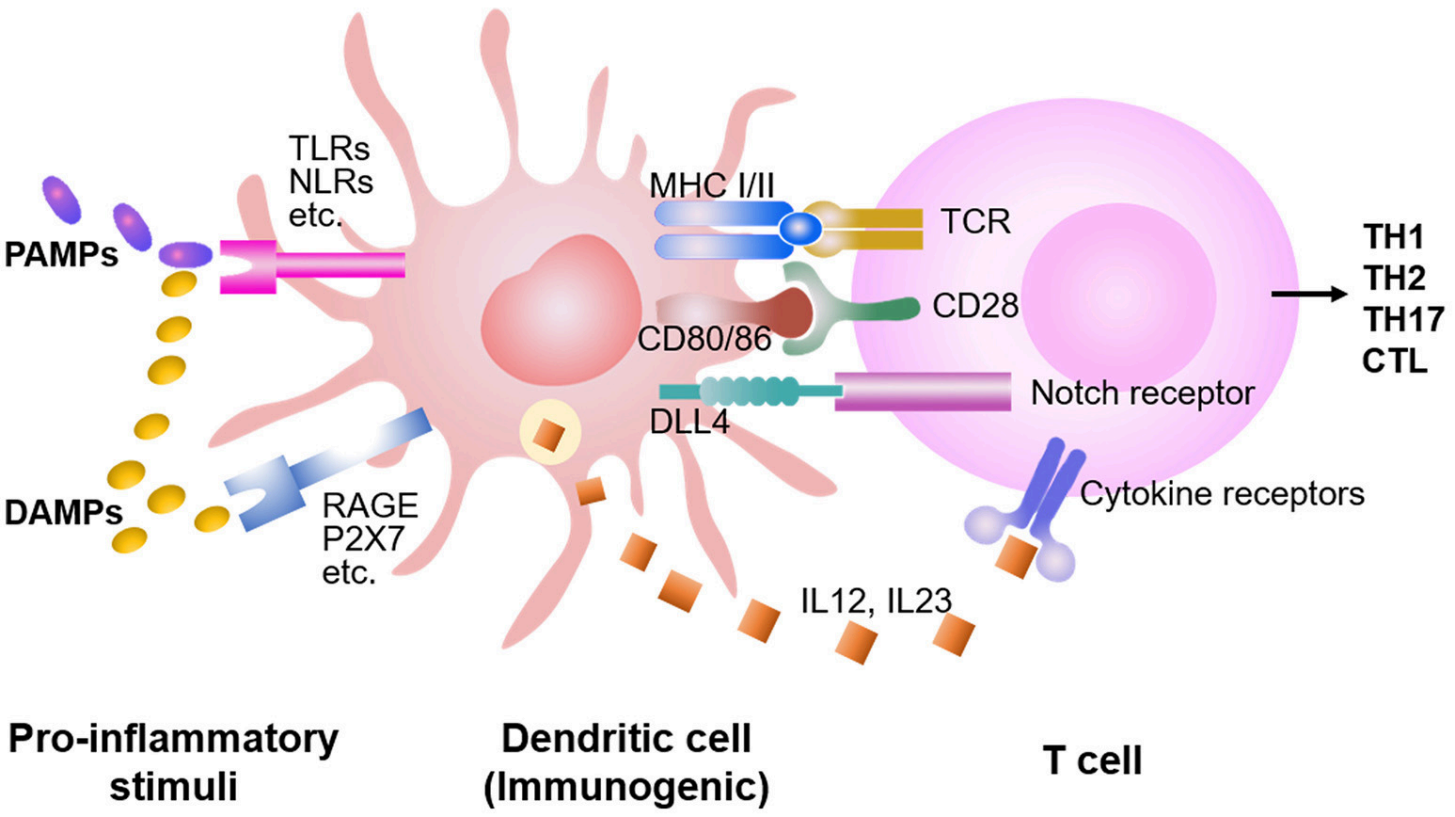
DC stimulation of allogeneic T cell responses. Preparative conditioning regimens before the allo-HSCT induce host tissue injuries, leading to the release of DAMPs and PAMPs. Consequently, DCs are activated by DAMPs and PAMPs through multiple receptors, capable to present antigens and prime the T cells. While DAMPs activate DCs mainly through TLRs (i.e., TLR 1-13), PAMPs activate DCs through RAGE, P2X7, etc., in addition to the TLRs. Both costimulatory molecules (e.g., CD28) and cytokines (e.g., IL-12, IL-23) synergize with the TCR signaling to promote proliferation and expansion of antigen-activated T cells. DCs also produce higher levels of Notch ligands (e.g., DLL1 and DLL4) to trigger Notch signaling in the T cell, instructing differentiation into distinct lineages of effector cells.

DCs produce multiple molecules capable of shaping allogeneic T cell responses (Figure 1). For example, IL-12 produced by DCs drives expansion and differentiation of antigen-activated T cells (13, 18, 27, 30, 62, 63). Donor BM cells lacking IL-12 p40 had significantly decreased capacity to promote effector differentiation and expansion in the mesenteric lymph nodes of mice receiving allogenic T cells. IL-12 derived from CD103^+^CD11b^−^ cDCs promoted IFN-γ production in host-reactive T cells (13). Notch signaling pathway is demonstrated as an important regulator of alloreactive T cell responses. Using a genetic approach, we reported that inhibition of pan-Notch receptor signaling in donor T cells significantly reduced severity and mortality of GVHD in mouse models (32). Notch-deprived T cells proliferated and expanded in response to alloantigen *in vivo*, but failed to produce inflammatory cytokines, including IFN-γ, IL-17, and TNF-α (31, 32). In a separate study, we further observed that host DCs expressing DLL4 (named DLL4^+^ DCs), one of the ligands of Notch receptors, had greater ability to stimulate the generation of alloreactive effector T cells that produced IFN-γ and IL-17 compared to DLL4^−^ DCs (64–66). Studies by Maillard et al. have shown that blockade of DLL4 could abrogate this effect and prevented GVHD while preserving anti-tumor activity (67, 68). Intriguingly, recent studies demonstrate that chemokine CCL19-expressing host cells, including both fibroblastic reticular cells and follicular DCs, were also the essential source of DLL4 for shaping alloreactive T cell response in mice subject to allo-HSCT (69). Collectively, DC-derived IL-12 and DLL4 are important for the generation of alloreactive effector T cells during GVHD.

### Activation of DCs After the Conditioning Regimens for Allo-HSCT

DCs express pattern recognition receptors (PRRs), such as Toll-like receptors (TLRs), and nod-like receptors (NLRs) to respond to pathogen-associated molecular patterns (PAMPs) (70–72). In addition, DCs are also capable to detect certain intracellular molecules, called damage-associated molecular patterns (DAMPs), that are released from cells stressed, damaged and/or dying in the local tissue (73). When PAMPs or DAMPs are present, DCs are stimulated to migrate to lymphoid tissues and present both antigen and costimulatory molecules to T cells (73–75). Preparative conditioning regimens for allo-HSCT, including high-dose chemotherapy and/or total body irradiation, cause host tissue injuries. This leads to the release of proinflammatory cytokines (e.g., TNF-α, IL-1β, and IL-6) as well as DAMPs and PAMPs (74, 76).

Both PAMPs and DAMPs activate DCs through stimulating TLRs (i.e., TLR1-13) (Figure 1) (1, 8, 76–78). TLR expression among DC subsets is heterogeneous: pDC mainly express TLR1, 7 and 9; CD8α^+^ DCs preferentially produce high levels of TLR3; whereas other cDC subsets express certain TLR subtypes but TLR9 (73–75, 79–85). Data from our studies and others suggested that Notch ligands DLL1 and DLL4 played non-redundant roles in activating Notch signaling to drive alloreactive T cell responses (32, 64–66, 68). LPS (TLR4 agonist) rapidly induces Dll4 expression in human and murine DCs (65, 66, 81–83). Combined stimulation of human DCs with LPS with TLR7 agonist R848 further increases the expression of DLL4 (65, 83). TLR3 is critical for presentation of viral double-stranded RNA (83, 86). Reddy and colleagues found that TLR3 stimulation enhanced GVL response without exacerbating GVHD in mice (52). These observations explain, at least in part, how different pro-inflammatory stimuli induce distinct types of immune responses.

## DC-mediated Donor T Cell Tolerance Against Host Tissues

Self-tolerance can be induced and maintained in different compartments of the immune system. During thymopoietic development, self-reactive T cells are clonally deleted in the thymus as a result of negative selection (8, 23, 36, 87). However, thymopoiesis is impaired during GVHD (88, 89), which is associated with generation of alloreactive T cells that mediate chronic-like GVHD in mice (90, 91). Considered as a supplemental mechanism to central tolerance, peripheral tolerance however, is important to prevent autoimmunity (8, 23, 36, 87). DCs are the crucial players mediating peripheral tolerance (27, 36, 37, 44, 87). Therefore, we will review the tolerogenic role of DCs in the context of allo-HSCT.

### cDCs

Both host and donor DCs may contribute to the induction of donor T cell tolerance against host tissues in mice undergoing allo-HSCT. Early studies by Teshima et al. reported that Flt3 ligand (Flt3L) treatment of recipient mice induced expansion of CD8α^+^ DCs that were poor stimulators of allogeneic T cells in cultures and had great ability to suppress donor T cell responses to host antigens *in vivo* (Table 1) (41). These Flt3L-treated recipient mice developed much less severe GVHD compared to untreated controls (41). However, whether these *in vivo* expanded CD8α^+^ DCs have direct effects on reducing GVHD was not examined in this study (41). Subsequent studies show that deletion of host CD11c^+^ cells in CD11c. DTR (diphtheria toxin receptor) transgenic recipient mice caused a strong increase in GVHD-related mortality (50). Since CD11c is also expressed on the surface of some macrophages (18, 19, 62), the possibility that DT treatment might delete CD11c^+^ macrophages that mediate immune suppression cannot be ruled out. Other studies examined the impact of deleting CD8α^+^ DCs on GVHD development in recipient mice lacking Batf3 (50), which is a transcription factor crucial for the generation of CD8α^+^ DCs and migratory CD103^+^ cDCs (92, 93). Recipient mice lacking Batf3 developed more severe GVHD compared to WT mice and marked increase of proliferative donor T cells (50). This finding is further supported independently by studies from Hill and colleagues (51), but not from Reddy's group (52). However, whether transfer of CD8α^+^ DCs may directly suppress GVHD in mice has never been reported. Thus, the exact DC subset induced upon Flt3L treatment capable to reduce GVHD has never been clearly addressed.

### pDCs

The important role of pDCs in modulating GVH response was initially shown in a mouse model of GVHD. Transfer of host-type CCR9^+^ pDCs inhibited GVHD in mice receiving MHC- or miHA-mismatched donor T cells (53). CCR9^+^ pDCs migrate to the GI tract through chemotaxis via their own chemokine receptor CCR9 and the ligand CCL25 in the environment. Upon stimulation with TLR9 agonist CpG ODNs, CCR9^+^ pDCs rapidly downregulate CCR9 from the original immature state and decrease the capacity to attenuate GVHD *in vivo* (53). Furthermore, precursor pDCs (pre-pDCs) were found to modulate GVHD in mouse models (55, 56). *In vivo* depletion of pre-pDCs using the antibody specific to PDCA-1, which is expressed on the surface of pDC lineage, significantly increased the severity of GVHD compared to recipient mice with intact donor pre-pDCs (55). Mechanistic analysis reveals that CCR9^+^ pDCs and pre-pDCs are capable to promote Treg expansion and function, as well as to suppress antigen-specific immune responses both *in vitro* and *in vivo* (55, 56). These observations identify the tolerogenic effect of pDCs on inducing donor T cells against host tissues.

### Molecular Mechanisms by Which DCs Induce Donor T Cell Tolerance

**Figure 2:**
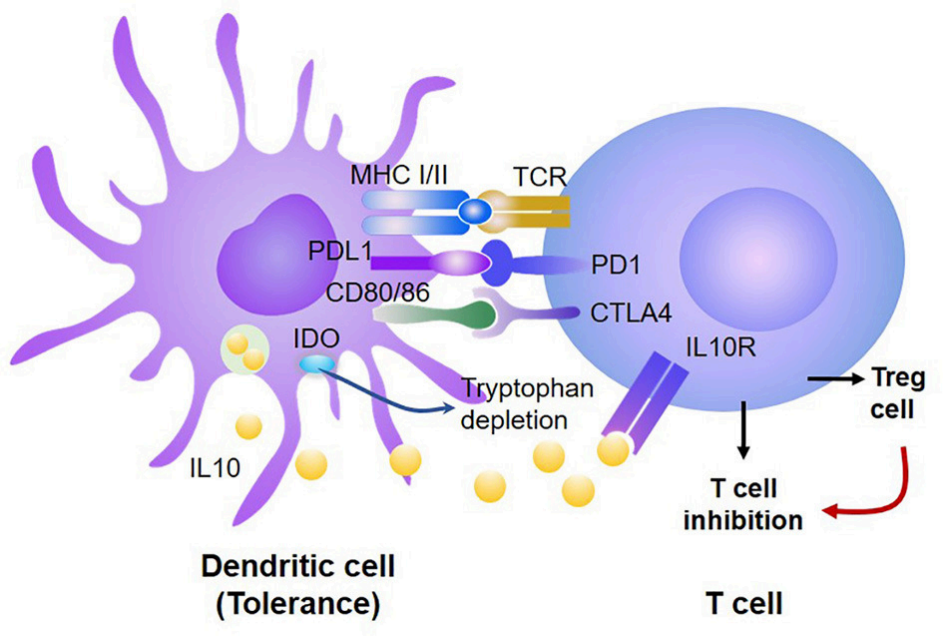
DCs induce donor T cell tolerance via both T-cell intrinsic and extrinsic mechanisms. Tolerogenic DCs produce high levels of PD-L1 and CD80/CD86, which, respectively, bind PD-1 and CTLA4, leading to inhibition of antigen-activated T cells and generation of Treg. Treg further suppress the proliferation and survival of those antigen-activated T cells. In addition, tolerogenic DCs produce high levels of IDO, which can inhibit antigen-reactive T cells and promote Treg expansion and function.

Emerging evidence indicate that the mechanism responsible for DC-induced peripheral T cell tolerance can be broadly classified into two categories: intrinsic and extrinsic (18, 23, 62, 87, 94). T cell intrinsic signal acts directly on the responding T cells, such as inhibition or deletion of specific T cells, while T cell extrinsic signal acts through additional cells or factors, such as Treg or suppressive cells (Figure 2).

Whether to induce immune activation or tolerance was initially correlated to the maturation state of DCs (62). Immature DCs generated from murine BM induced T cell unresponsiveness *in vitro* and prolonged cardiac allograft survival (43, 95). The immune tolerance induced by immature DCs was associated with their low expression of CD40 (which is essential to activation of CD4^+^ T cells) and the capacity to produce high levels of IL-10 (which inhibits T cell response). Probst et al. reported that resting DCs induced peripheral CD8^+^ T cell tolerance through activating the inhibitory signals PD-1 and CTLA4 on the T cell via PD-L1 and CD80/86, respectively (96–104). Under physiological conditions, these inhibitory molecules keep autoreactive T cells in check without causing autoimmunity. Blocking either PD-1 or CTLA4 abrogated CD8^+^ T cell tolerance induction and enhanced T cell priming while blocking both resulted in an synergistic effect on inducing CD8^+^ T cell tolerance (96). These observations suggest that DC-derived PD-L1 may promote T cell tolerance through triggering the T-cell intrinsic mechanism.

Treg and suppressive cells (e.g., myeloid-derived suppressive cells) play crucial roles in establishing and maintaining peripheral tolerance and are known to be important for reducing GVHD (105–108). Waller and colleagues have demonstrated that transfer of donor BM pre-pDCs attenuated GVHD in mice (56). They identified that donor pDCs activated donor T cells to produce IFN-γ, which then enhanced pDC synthesis of indoleamine 2,3-dioxygenase (IDO). Increased production of IDO by pDCs altered the balance between donor Treg and alloreactive effector T cells, thereby limiting the severity of GVHD (56). Other studies showed that GVH reaction also impaired the antigen presentation function of *de novo* generated donor cDCs, leading to dramatically decreased Treg expansion and function, leading to severe chronic GVHD (109). These observations suggest that reconstitution of tolerogenic DCs from engrafted donor hematopoietic cells may be crucial for preventing the occurrence of severe GVHD.

## Reconstitution of Donor DCs After Allo-HSCT

Clinical studies indicated that impaired reconstitution of donor DCs correlates with the occurrence of severe GVHD (110–115). Wingard and colleagues examined the number of donor DCs in the circulating peripheral blood from a group of 50 allo-HSCT patients. They found that low number of circulating DCs was not only associated with significantly increased risk of relapse and acute GVHD, but also predicative of patient death after allogeneic HSCT (110). Notably, clinical studies from 39 children with allo-HSCT indicated that while normal cDC numbers were observed by 300–400 days after transplantation, pDC numbers were always lower than those of age-matched control patients during the entire follow-up period of up to 7 years (112). In contrast, patients with high pDC recovery profile often had improved overall survival (114). Data from preclinical studies also showed a marked deficit in all lineages of DCs (CD8^+^ DCs, CD11b^+^ DCs, and pDCs) in GVHD compared with non-GVHD mice (55, 109, 116, 117). Thus, the recovery of DCs from engrafted HSPCs in allo-HSCT patients may predict the occurrence of severe GVHD and non-relapse mortality.

### Donor DCs Mediate Protective Immunity

DCs are critical for eliciting T cell immune responses, protecting the host against viral infection (18, 19, 118). Viral infection remains a major challenge for the success of allo-HSCT. Clinical studies have shown that after allo-HSCT, patients with lower numbers of circulating peripheral blood DCs often have increased risk of infections (1, 110). Cytomegalovirus (CMV) is a major cause of post-transplant mortality in patients subject to allo-HSCT, with ~25% of CMV-seropositive recipients developing CMV-related disease within 3 months after transplantation (119–121). It is well-established that induction of adaptive T cell immunity is critical to control CMV replication and resolve viral reactivation-mediated disease (100, 122, 123). cDCs are essential to the generation of effector T cells reactive to CMV, especially during a primary response. However, GVHD induces a profound DC defect that leads to a failure in the generation of CMV-specific CD8^+^ T-cells and dramatically decreased antiviral immunity (116). Collectively, improving the reconstitution of DCs following allo-HSCT may represent an effective strategy to re-establish the protective immunity in the recipient. Since pDCs produce high levels of IL-12 and IFN-α upon activation (29, 124–126), improving pDC recovery after allo-HSCT may also provide efficient antiviral immunity.

### GVHD Impairs the Generation of DC Progenitors

GVHD-associated inflammatory responses may influence the reconstitution of donor DCs via a complex mechanism. DCs developed from HSPCs through successive steps of lineage commitment and differentiation: HSCs → multiple potent progenitors (MPP) → common DC progenitors (CDP) → cDCs and pDCs (22, 42, 125, 127–132). Inflammatory cytokines, such as TNF-α and IFN-γ, directly inhibit the proliferation of HSPCs and their generation of DCs (117). In addition, Matsushima and colleagues have shown that GVHD induced damage to the BM niche, leading to dramatically decreased hematopoiesis, including the reduction of CDP (133). However, the specific cellular component(s) within the niche that are responsible for the generation of DC progenitors have yet to be determined.

### Transcriptional Regulation of DC Development

The generation of DCs is regulated by a group of functionally distinctive transcription factors (TFs). Analysis of gene-targeted mice has identified many critical TFs in DC development. Some of these TFs, such as Pu.1 and Stat3, influence the generation of all DC subsets. HSPCs lacking Pu.1 showed defective DC differentiation potential (134, 135). Targeted deletion of Stat3 impaired the generation of both cDCs and pDCs *in vivo* (128, 136). Thus, both Pu.1 and Stat3 are pioneer TFs in the regulation of DC commitment and differentiation from MPP (42, 128, 130).

DC subset-specifying TFs are required for committed CDP to become functionally distinct DC lineages (42, 127, 128, 130). For example, Batf3 has a non-redundant role in CD103^+^ cDC development and partial effect on inducing CD8α^+^ DCs in lymph organs (52, 92, 127). Irf8-deficient animals lack spleen-resident CD8α^+^ cDCs and nonlymphoid tissue CD103^+^ cDCs (42, 127, 137). Other TFs, such as Irf4, Klf4, Notch2, and Relb, also play important roles in the regulation of other types of cDCs localized in different tissues (94, 138). Among them, Irf4 is required for cDCs to prime CD4^+^ T cells and promote Th17 differentiation in both the lung and intestine (60, 139). In addition, several TFs, such as Tcf4, Irf8, and Spib, are known to regulate pDC differentiation (140–142). The absence of Tcf4 leads to the loss of pDCs in mice (142).

There are limited number of studies investigating how GVH reactions influence the expression and function of these TFs required for DC development. Notably, a recent study revealed that inflammation cascades in GVH reaction favor the development of CD103^+^D11b^−^ DCs in the GI tract (42), which require the presence of functional Irf4 (93). These data indicate that distinct TFs in DCs and their progenitors may have different susceptibility to the regulatory effect of inflammatory environments. This may result in a skewed expression and activation of transcriptional programs, promoting the generation of specific subset(s) of DCs and feed-forward action on alloreactive T cell responses during the GVHD progression. Delineating the mechanisms underlying this dysregulated donor DC reconstitution during GVH reaction will be important for understanding the pathophysiology of GVHD and the development of effective treatments for the disease.

## DC Modulation of Alloimmunity

Manipulation of DC precursors in the HSPC graft may facilitate the establishment of a balance between GVHD and GVL effects (2, 9, 16, 34, 35, 56). Preclinical studies have shown that transfer of donor pre-pDCs derived from donor mice treated with Flt3L induced markedly augmented GVL activity of donor T cells without aggravating GVHD (56). These donor pre-pDCs persisted long in that they expanded *in vivo* for 2 weeks after transplantation (56). These findings perfectly supported the clinical value of donor DCs in modulating alloimmunity to improve the efficacy and safety of allo-HSCT.

### Use of Tolerogenic DCs to Reduce GVHD

The capacity of DCs to induce tolerance has led to numerous therapeutic studies using these cells in an effort to control harmful immune responses in models of allograft rejection, GVHD and autoimmune disorders (18, 27, 34, 36, 37, 62, 87, 143). While transfer of immature CCR9^+^ pDCs reduced GVHD, transfer of mature donor pDCs did not as expected (144). Furthermore, transfer of highly purified immature pDCs derived from donors was technically challenging and typically required *in vivo* expansion step to generate the number enough for modulating GVH reaction *in vivo* (53, 56, 144).

With this technical bottle neck, many studies had to assess the therapeutic effect of *ex-vivo*-generated DCs. Tolerogenic DCs were tried to be generated through propagating human monocytes *in vitro* in the presence of various agents, such as IL-10, Vitamin D3, and immunosuppressive drugs (e.g., dexamethasone and rapamycin) (34, 36, 37, 87, 143). Yet, none of the approaches generated the best clinically applicable DCs with the expected tolerogenic capacity to modulate alloreactive T cells (34, 36, 37, 87, 143). Reddy and colleagues report that upon pre-treatment with the HDAC inhibitor SAHA, these moDCs produced high levels of IDO and promoted Treg expansion and function *in vivo*, leading to attenuated GVHD in mice (54). These findings indicate that targeting epigenetic regulators in DCs may prove to be an effective strategy to induce the generation of DCs with tolerogenic properties for reducing GVHD.

### DC Induction of GVL Effects After Allo-HSCT

Emerging evidence indicated that DCs were required for optimal GVL response without aggravating GVHD. Reddy and colleagues report that as compared to allogeneic wild-type (WT) hosts, allogeneic Batf3-deficient recipient mice developed severe GVHD but with significantly reduced GVL response (52). This indicates the importance of CD8α^+^ DCs in GVL response. Indeed, co-transfer of WT host-type spleen DCs (which contain CD8α^+^ DCs) and T cells into allogeneic B2m^−/−^ recipients, which are functionally deficient in antigen presentation, induced a significant CD8^+^ T cell-mediated GVL response, leading to prolonged survival of recipients without tumor. In contrast, all of the B2m^−/−^ mice receiving Batf3^−/−^ spleen DCs, which lack CD8α^+^ DCs (92), died from tumor despite the presence of other DC subsets (52). This confirms the crucial role of CD8α^+^ DCs in eliciting anti-tumor immunity. However, these experiments did not examine the direct effect of CD8α^+^ DCs on T cell-mediated GVL response.

In human recipients of unrelated donor BM grafts, but not granulocyte colony-stimulating factor (G-CSF)-mobilized peripheral blood grafts, a higher number of donor pDCs is associated with increased survival and reduced GVHD (145). Data from experimental studies indicate that transfer of pDC-enriched BM grafts preserved GVL effects without aggravating GVHD in mice (56, 146). pDCs produce high levels of IFN-α and IL-12 (147, 148), cytokines important to promote differentiation and expansion of antigen-specific effector cells (149). In these studies, Waller and colleagues have demonstrated that *in vivo* administration of Flt3L to donor mice induced 5-fold increase in pDC content without significant changes in the number of HSCs, T cells and B cells. Most importantly, transfer of pDC-enriched BM graft from Flt3L-treated donors decreased GVHD while retaining GVL effects in allogeneic recipient mice (146).

We have recently established a novel platform to produce Dll4^+^ DCs from murine BM using Flt3L and TLR agonists (64). Upon allogeneic Dll4^+^ DC stimulation, CD4^+^ naïve T cells underwent effector differentiation and produced high levels of IFN-γ and IL-17 *in vitro*, depending on Dll4 activation of Notch signaling. Adoptive transfer of these Dll4^+^ DC-induced T cells eliminated leukemic cells without causing severe GVHD, leading to significantly improved survival of leukemic mice undergoing allo-HSCT. This strategy may potentially improve the anti-leukemic response after HSCT and overcome some barriers to the GVL response such as high disease burden and pharmacologic immunosuppression (64). Since DC activation of naïve T cells allows them to be primed with antigens, Dll4^+^ DCs loaded with leukemia-associated antigens may facilitate the selective expansion of leukemic cell-reactive T cells and specifically boost the anti-leukemia activity.

## Conclusion

While traditional therapies have been targeting T cells, extensive research in murine HSCT models convincingly showed the ability of DCs to preserve GVL response without aggravating GVHD. Targeting donor DCs *in vivo* or *ex vivo* may potentially subvert alloreactive T cell responses and reduce GVHD (53). Given the role of DCs in maintaining Treg after allo-HSCT (56, 109), co-transfer of tolerogenic DCs and Treg could be more effective on reducing GVH reactions *in vivo*. A randomized Phase I study has shown the safety of infusing the host tolerogenic DCs into diabetes patients (150). It will be interesting to test whether these *ex vivo* generated tolerogenic DCs given in the peri-transplant period may prevent GVHD while preserving GVL effects.

One major challenge is to produce large number of donor-type tolerogenic DCs that can persist sufficient time to execute their function following adoptive transfer to modulate alloimmunity. We propose that donor-type DCs have several advantages compared to host-type DCs. For example, donors are healthy, and their hematopoietic system is not compromised by accompanied disease state and treatment conditions. Furthermore, available data from both clinical and pre-clinical studies suggest that donor-derived pDCs have potent capability to modulate GVH reactions (55, 56, 145, 146). These data provide a proof of concept that *in vivo* administration of pDCs is promising for enhancing GVL response without causing severe GVHD.

Most recent studies have shown that the fate of pDCs is determined early at the stage of HSCs (127, 130, 136, 151–153). This suggests that induction of tolerogenic DCs should start from the HSPC stage. Better understanding how the fate of tolerogenic DCs are determined and regulated may have significant implication in the production of DCs for efficiently modulating alloimmunity.

## Author Contributions

HY, YT, YW, SM, and YZ collected all materials for reviewing and wrote the review.

### Conflict of Interest Statement

The authors declare that the research was conducted in the absence of any commercial or financial relationships that could be construed as a potential conflict of interest.
